# Systematic Review and Meta-Analysis on the BeGraft Peripheral and BeGraft Peripheral PLUS Outcomes as Bridging Covered Stents in Fenestrated and Branched Endovascular Aortic Repair

**DOI:** 10.3390/jcm14155221

**Published:** 2025-07-23

**Authors:** George Apostolidis, Petroula Nana, José I. Torrealba, Giuseppe Panuccio, Athanasios Katsargyris, Tilo Kölbel

**Affiliations:** 1 German Aortic Centre, Department of Vascular Medicine, University Medical Center Eppendorf, 20251 Hamburg, Germany; petr.nana7@hotmail.com (P.N.); jitorrealba@gmail.com (J.I.T.); giuseppe.panuccio@gmail.com (G.P.); tilokoelbel@googlemail.com (T.K.); 2 Department of Vascular and Endovascular Surgery, General Hospital Nuremberg, Paracelsus Medical University, 90419 Nuremberg, Germany; kthanassos@yahoo.com

**Keywords:** BeGraft, target vessel, bridging stent, fenestrated endovascular repair, branched endovascular repair

## Abstract

**Background/Objective**: Bridging stent optimal choice in fenestrated and branched endovascular aortic repair (f/bEVAR) is under investigation. This systematic review and meta-analysis studied the outcomes of the BeGraft peripheral and peripheral PLUS as bridging stents in f/bEVAR. **Methods**: The methodology was pre-registered to the PROSPERO (CRD420251007695). Following the PRISMA guidelines and PICO model, the PubMed, Cochrane and Embase databases were searched for observational studies and randomized control trials, in English, from 2015 to 2025, reporting on f/bEVAR patients using the second-generation BeGraft peripheral or the BeGraft peripheral PLUS balloon expandable covered stent (BECS; Bentley InnoMed, Hechingen, Germany) for bridging. The ROBINS-I assessed the risk of bias and GRADE the quality of evidence. Target vessel technical success, occlusion/stenosis, endoleak Ic/IIIc, reintervention and instability during follow-up were primary outcomes, assessed using proportional meta-analysis. **Results**: Among 1266 studies, eight were included (1986 target vessels; 1791 bridged via BeGraft); all retrospective, except one. The ROBINS-I showed that seven were at serious risk of bias. According to GRADE, the quality of evidence was “very low” for primary outcomes. Target vessel technical success was 99% (95% CI 98–100%; I^2^ = 12%). The mean follow-up was 20.2 months. Target-vessel instability was 3% (95% CI 2–5%; I^2^ = 44%), occlusion/stenosis was 1% (95% CI 1–4%; I^2^ = 8%) and endoleak Ic/IIIc was 1% (95% CI 0–3%; I^2^ = 0%). The estimated target-vessel reintervention was 2% (95% CI 2–4%; I^2^ = 12%). Celiac trunk, superior mesenteric and renal artery instability were 1% (95% CI 0–16%; I^2^ = 0%;), 1% (95% CI 0–5%; I^2^ = 14%) and 4% (95% CI 2–7%; I^2^ = 40%), respectively. **Conclusions:** The BeGraft peripheral and peripheral PLUS BECS performed with high technical success and low instability when used for bridging in f/bEVAR. Cautious interpretation is required due to the very low quality of evidence.

## 1. Introduction

Bridging covered stent (BCS) selection in fenestrated and branched endovascular aortic repair (f/bEVAR) is a field of significant interest, as target vessel adverse events have been shown to affect up to 10% of patients during the initial 30 days after f/bEVAR, while 25–50% of them may need further intervention [1,2]. Endoleaks seem to affect the clinical success of f/bEVAR, leading to a lower sac regression rate at 12 months [2]. However, aneurysm rupture during follow-up is a rare phenomenon [1]. While balloon-expandable covered stents represent the BCS of choice for fEVAR, BCS selection in bEVAR relies on a patient’s anatomy, surgeon’s preference and device availability while the available literature seems to be rather inconclusive regarding optimal stent selection [3,4].

The first generation of the BeGraft peripheral did not perform well, being an independent predictor for target vessel-related endoleaks and reinterventions after f/bEVAR [5,6]. The higher rate of target vessel instability seemed to have a direct correlation to fractured first-generation BeGrafts [6]. This led to the launch of the second-generation BeGraft peripheral in 2015, which was followed in 2017 by the launch of the BeGraft peripheral PLUS [7]. The second-generation BeGraft peripheral stent is a balloon-expandable covered stent, constituting of a single layer of expanded polytetrafluoroethylene (ePTFE) bonded to a cobalt chromium metal stent [8]. When compared to its first-generation predecessor, it is characterized by increased thickness of ePTFE and increased width of stent connectors [9]. This stent demonstrates high flexibility and conformability with low bending stiffness, owing to its single layer of ePTFE, minimizing wall stress and neointimal hyperplasia, as well as cyclic stent deformation and metal fatigue [8]. BeGraft peripheral PLUS has been introduced as an enhanced alternative and has been preferably used in bridging of target vessels during bEVAR [7]. It constitutes a double-covered stent platform providing enhanced durability and superior pull-out and shear-stress force resistance, without compromising flexibility [8,10]. Single center analyses and in vitro tests showed favorable outcomes in term of conformity in target vessel anatomy, without stent fracture even after flaring, and high rates of target vessel patency and freedom from instability [7,8,10,11]. However, meta-analytic data on the outcomes of BeGraft balloon-expandable covered stents as BCS in f/bEVAR are lacking.

The aim of this systematic review and meta-analysis was to assess the second-generation BeGraft peripheral and BeGraft peripheral PLUS outcomes as BCS in f/bEVAR procedures at 30 days and during follow-up.

## 2. Materials and Methods

### 2.1. Eligible Studies

This systematic review and meta-analysis was conducted based on the Preferred Reporting Items for Systematic Reviews and Meta-analyses (PRISMA) guidelines [12]. Methodological details ensuring the integrity of the study were predefined and registered to the PROSPERO (CRD420251007695). Prospective and retrospective observational cohort studies, as well as any randomized control trial, published in English from 1 January 2016 to 2025, reporting on patients managed with fenestrated or branched endovascular aortic repair (f/bEVAR) using the second-generation BeGraft peripheral or the BeGraft peripheral PLUS balloon expandable covered stent (Bentley InnoMed GmbH, Hechingen, Germany) were considered eligible. The search start date restriction was applied since the second-generation BeGraft peripheral stents became available in late 2015. Studies had to report on more than ten patients managed with f/bEVAR for complex aortic aneurysms and include at least ten BeGraft peripheral/BeGraft peripheral PLUS as BCS. The patients could have been treated with custom-made or physician-modified fenestrated or branched aortic devices and any commercially available off-the-shelf branched device.

Studies reporting on patients managed with parallel graft techniques were excluded. The type of complex aortic aneurysm (thoracoabdominal, juxtarenal or pararenal), the number of revascularized target vessels per case and the duration of the available follow-up were not taken under consideration as exclusion criteria. Studies reporting exclusively on other balloon expandable stents as well as studies that did not provide extractable data on the BeGraft peripheral/BeGraft peripheral PLUS were also excluded. Among studies with potential overlapping populations, the most recently published study or the one presenting with the largest cohort was included in the analysis.

### 2.2. Search Strategy

The PICO [Patient; Intervention; Comparison; Outcome (Table S1)] model was used to plan and conduct a systematic search of the available English literature using PubMed, Cochrane Library and Embase (via Ovid) databases [13]. The final search was performed on 15 April 2025. The following search terms, including Expanding Medical Subject Heading (MeSH terms), were used in various combinations (Table S2): “thoracoabdominal aortic aneurysm”, “juxtarenal aortic aneurysm”, “pararenal aortic aneurysm”, “fenestrated endovascular aortic repair”, “branched endovascular aortic repair”. No terms describing outcomes were used, to avoid limiting the sensitivity of the search. An initial deduplication process was performed both by an automation tool (Endnote, Version 21, Clarivate Analytics, Philadelphia, PA, USA) and by hand. Available records after deduplication were reviewed and assessed for eligibility by two authors, initially based on their title and abstract and, subsequently, based on their full text (G.A. and P.N.). Discrepancies were resolved after discussion with the senior author (T.K.).

### 2.3. Data Extraction

Data extraction was performed using a standardized Excel file, created and pilot-tested by two authors (G.A. and P.N.). This was used to extract information on general study characteristics (authors, publication year, study design, timespan, participating centres), as well as baseline information on patient cohorts (number of patients, age, sex) underlying aortic disease [juxtarenal, pararenal or thoracoabdominal (categorized according to Crawford classification) aneurysm], number of target vessels, number of BeGraft peripheral or BeGraft peripheral PLUS balloon expandable stents used for bridging, type and platform of endografts. Additionally, information on outcome definitions and patient inclusion criteria were collected. Regarding the outcomes of interest (30-day, follow-up and target vessel specific), event counts on technical success, occlusion/stenosis, endoleak type Ic/IIIc, reinterventions and instability were extracted. Two authors (G.A. and P.N.) performed data extraction. Any discrepancy was resolved after discussion with the senior author (T.K.).

### 2.4. Quality Assessment

The ROBINS-I tool for non-randomized observational studies was applied for the individual study risk of bias assessment. The ROBINS-I is used to evaluate seven domains of potential risk of bias, providing a score (“low”, “moderate”, “serious” or “critical”), to achieve the maximum transparency and reliability of the conducted evaluation [14]. The Grading of Recommendations Assessment, Development and Evaluations (GRADE) was used to evaluate the overall quality of evidence for each one of the main outcomes, by assessing seven key components, including the risk of bias, inconsistency, indirectness, imprecision, large effect, dose response and confounder control [15]. Both assessments were performed by two independent investigators (G.A. and P.N.), and any discrepancy was resolved after discussion with a senior author (T.K.).

### 2.5. Definitions

Definitions were accepted as described in the included studies due to considerable heterogeneity between studies regarding the reported results. According to the currently available Society for Vascular Surgery reporting standards [16], the target vessel technical success was defined as the composite of successful target vessel catheterization and BCS deployment with a patent-intended target vessel and absence of type Ic or IIIc endoleak, extending beyond 30 days [16]. Target vessel instability was considered any composite outcome, including death, aortic rupture, occlusion or component separation related to side branch complication or any target vessel-related secondary intervention [16]. Accordingly, endoleak Ic was considered the inadequate distal sealing of the BCS inside the target vessel, while endoleak IIIc was attributed to disconnection or apposition failure [16].

### 2.6. Outcomes

The primary outcomes were technical success, as well as occlusion/stenosis, endoleak Ic/IIIc, reintervention and target vessel instability rate during the available total follow-up period. The same outcomes were evaluated at 30-days as well as per type of target vessel and were considered as secondary outcomes. A sensitivity analysis was performed regarding the primary outcomes of vessels targeted through a fenestration.

### 2.7. Statistical Analysis

Data regarding the outcomes of interest were extracted as event counts, while incidence proportion along with 95% confidence intervals was calculated for each individual study and synthesized through meta-analysis of single proportions. Only integer proportions are reported, complying with our data qualitative and quantitative characteristics. In case of rates between 0 and 1%, one additional significant digit was presented to demonstrate the non-zero result. Between-study heterogeneity was statistically assessed through tau squared and I^2^ statistics, which were presented through forest plots, along with individual studies and syntheses’ effect estimates. A generalized linear mixed-model method based on logit transformation through the ‘metaprop’ function was used to calculate the summary proportions along with their 95% confidence intervals [17]. The method was preferred due to small sample size, as well as event count of included studies. Due to probable intrinsic heterogeneity between individual studies samples, irrespective of heterogeneity statistics’ values, the random-effects model was chosen to be presented. A maximum likelihood estimator was used for tau squared and the Knapp and Hartung method was used to calculate confidence intervals for the overall estimate. Publication bias was assessed through the Egger’s test and visualized in funnel plots, if at least 10 studies were included in the quantitative synthesis. No imputation methods were applied for missing values. A continuity correction of 0.5 was only applied for individual study results. When presented as a summary, categorical data regarding patients’ characteristics were reported as event counts, while continuous data were expressed as weighted means and corresponding 95% confidence intervals (calculated by an inverse variance weighting-based method through ‘metamean’ function). Additionally, when medians with a range or interquartile range were reported, conversion to mean values and corresponding standard deviations was applied [18,19]. Statistical analyses and corresponding plots were performed with RStudio (Version 2024.12.0+467; Integrated Development Environment for R, Posit Software, PBC, Boston, MA, USA).

## 3. Results

### 3.1. Study Selection and Risk of Bias

**Figure 1:**
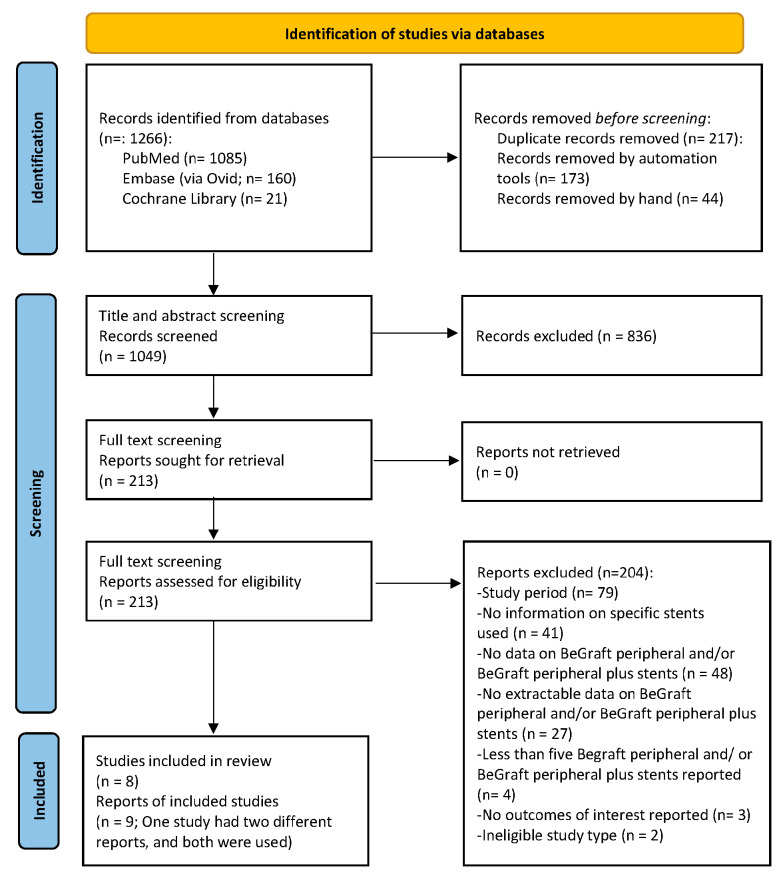
PRISMA 2020 flow chart depicting the study selection process. Eight studies (nine study reports) have been included in the present systematic review and meta-analysis [7,8,9,11,20,21,22,23,24].

After a prespecified systematic search of the literature, 1266 study reports were retrieved and scanned for eligibility. The stepwise study selection process resulted in eight studies (with nine study reports) being included in the qualitative and quantitative synthesis (Figure 1) [7,8,9,11,20,21,22,23,24]. Regarding the case of Clough et al. and Spear et al., both studies reported results on the same cohort, with the later one providing further insight on early outcomes [9,20]. All studies were observational; one of them was prospective [8], while seven were retrospective [7,11,20,21,22,23,24]. The main characteristics of the included studies are shown in Table 1.

The risk of bias evaluation with the ROBINS-I tool [14] revealed that seven out of eight studies were at serious risk [7,8,11,20,21,22,23] and one at moderate risk [24]. Bias was mainly attributed to insufficient addressing of confounding factors and lack of appropriate analyses, postoperative information used to classify the intervention, observational type, as well as extent and handling of missing data (Figure S1). According to the GRADE assessment, the quality of evidence was characterized as “very low” for all primary outcomes, explained by the observational type of included studies and the risk of bias assessment [15]. GRADE and “Summary of evidence” are provided in Tables S3 and S4.

### 3.2. Patients and Target Vessels’ Cohort

All patients of the included studies were managed with either fenestrated or branched devices [7,8,11,20,21,23,24]. There was one study where both were used [22]. Cook devices have been, mainly, used including custom-made branched or fenestrated (Zenith platform), off-the-shelf (“T-branch”) or physician-modified (Cook Medical LLC, Bloomington, IN, USA) endografts [7,8,11,20,21,22,23]. Moreover, the Anaconda custom-made fenestrated device was used (Terumo Aortic, Bolton Medical Inc., Sunrise, FL, USA) in two studies [21,24] and the “E-nside” off-the-shelf branched endograft (Artivion EMEA GmbH, Kennesaw, GA, USA) in one study [7] (Table 1).

There were 678 patients (567 males; 84%). The mean age was 73.3 (95% CI 71–75.6) years. Only 27 patients were treated urgently. A custom-made fenestrated device was implanted in 443 patients, and a physician-modified fenestrated device was implanted in 24 patients. Regarding the rest of the patients, 165 were managed with custom-made devices incorporating both branches and fenestrations, 39 were managed with custom-made branched devices and seven were managed with off-the-shelf branched devices. Among the included studies, only two provided extractable data on urgent/emergent cases. In Becker et al. [11], 20 urgent cases were managed; nine of them addressed a rupture. In Abisi et al. [7], seven cases were managed emergently, either due to symptoms or “leaking”. Mancuso et al. [24] also included urgent cases but information was not extractable explicitly for patients treated with BeGraft peripheral stents.

In total, 1986 renovisceral vessels were targeted and 1740 were bridged with 1791 BeGraft balloon-expandable covered stents, 1329 BeGraft peripheral and 462 BeGraft peripheral PLUS. All BeGraft peripheral stents were used to bridge fenestrations, except from one that was used to bridge a branch (reported in Katsargyris et al. [22]), while all BeGraft peripheral PLUS stents were used to bridge branches, except from 17 that were used to bridge fenestrations (reported in Katsargyris et al. and Mancuso et al. [22,24]). Information on patients’ characteristics is included in Table 2.

### 3.3. BeGraft Peripheral and BeGraft Peripheral PLUS Target Vessel-Related Outcomes

#### 3.3.1. Technical Success and Adjunctive Procedures

**Figure 2:**
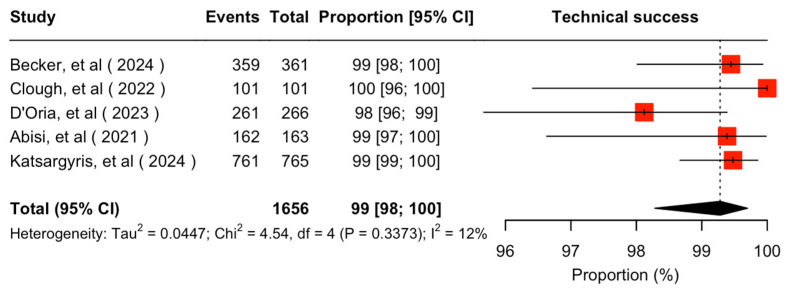
Forest plot for technical success rate [7,11,20,21,22].

Target vessel-related technical success was extractable in five studies [7,9,11,21,22]. The cumulative technical success rate was 99% (95% CI 98–100%; I^2^ = 12%) (Figure 2). In the rest of the included studies, technical success was not considered extractable [8,23,24]. Several target vessel-related adjunctive intraoperative procedures were reported in the included studies consisting mainly of additional stent implantation for various reasons (endoleak, angulation, dissection, stent fracture, kinking, dislodgement or rupture) as well as target vessel embolization. Technical success definition along with target vessel-related adjunctive intraoperative procedures per study are described in Table 3.

#### 3.3.2. Thirty-Day Outcomes

Meta-analyses on 30-day outcomes included five studies [7,11,20,21,23]. The estimated target vessel instability rate was 0.2% (95% CI 0–7%; I^2^ = 6%; Figure S2A). The cumulative occlusion/stenosis rate was 0.3% (95% CI 0–4%; I^2^ = 0%; Figure S2B). Two renal arteries and one superior mesenteric artery occluded or presented a stenosis. One renal artery was successfully recanalized through aspiration thrombectomy and implantation of an additional BeGraft peripheral with restoration of the initially deteriorating renal function [11]. An additional renal artery failed to be recanalized [20]. A superior mesenteric artery (SMA) dissection with subsequent bowel ischemia was managed with relining and bowel resection. The authors did not consider it a stent-related event [20].

The estimated endoleak Ic/IIIc rate was 0% (95% CI 0–1%; I^2^ = 0%; Figure S2C). No endoleaks were reported during the first 30 days. Ultimately, the cumulative reintervention rate at 30 days was 1% (95% CI 0–4%; I^2^ = 9%; Figure S2D). Apart from the reinterventions for occlusion/stenosis mentioned earlier, there was one reintervention due to an ileal artery branch rupture [20], as well as two prophylactic reinterventions to avoid endoleak or dislodgement due to inadequate BCS distal landing; however, these were not considered events of target vessel instability [7].

#### 3.3.3. Follow-Up Outcomes

**Figure 3:**
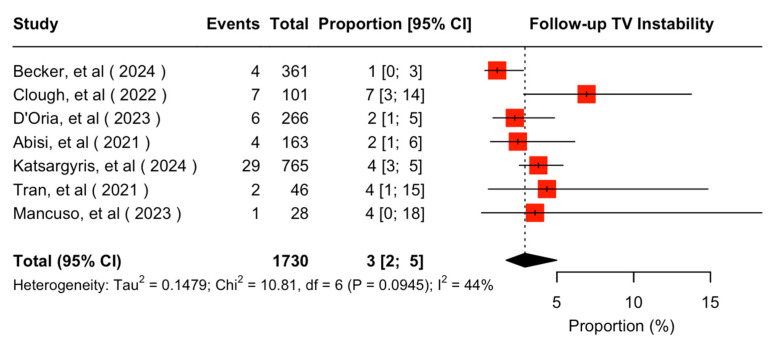
Forest plot for target vessel instability rate during follow-up [7,8,11,20,21,22,24].

**Figure 4:**
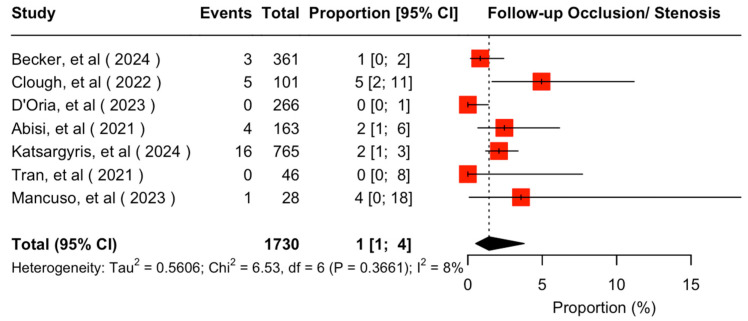
Forest plot for target vessel occlusion/stenosis rate during follow-up [7,8,11,20,21,22,24].

**Figure 5:**
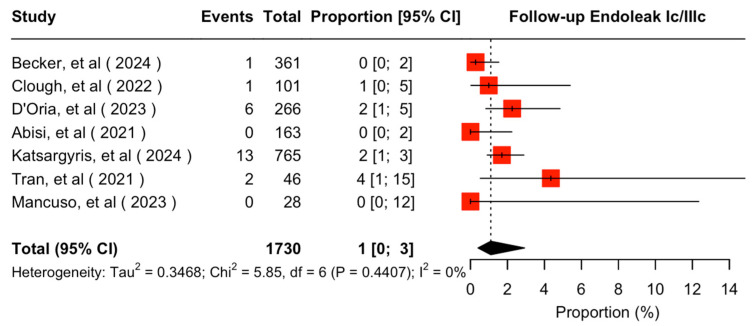
Forest plot for endoleak Ic/IIIc rate during follow-up [7,8,11,20,21,22,24].

**Figure 6:**
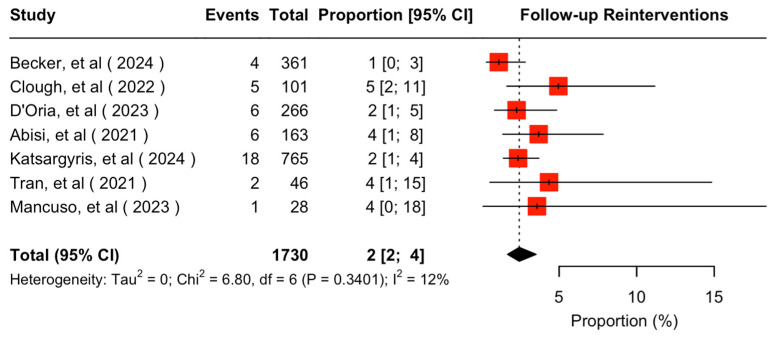
Forest plot for reintervention rate during follow-up [7,8,11,20,21,22,24].

The mean available follow-up was 20.2 (95% CI 13.7–26.7) months. Data on follow-up outcomes were available and extractable in seven studies [7,8,11,20,21,22,24]. The estimated target vessel instability was 3% (95% CI 2–5%; I^2^ = 44%; Figure 3). The cumulative target vessel occlusion/stenosis rate was calculated at 1% (95% CI 1–4%; I^2^ = 8%; Figure 4), while the estimated endoleak Ic/IIIc rate was 1% (95% CI 0–3%; I^2^ = 0%; Figure 5). The estimated target vessel-related reintervention rate was 2% (95% CI 2–4%; I^2^ = 12%; Figure 6).

#### 3.3.4. Follow-Up Outcomes per Type of Target Vessel

The same outcomes were investigated per target vessel type. Data were available and extractable in six studies for all outcomes [7,8,11,20,21,22], except from target vessel-related reinterventions which could be extracted only from five studies [7,8,11,20,21]. There were 344 celiac trunks, 439 superior mesenteric arteries and 921 renal arteries included in the analyses.

The estimated celiac trunk instability rate was 1% (95% CI 0–16%; I^2^ = 0%; Figure S3A). The cumulative occlusion/stenosis rate was 1% (95% CI 0–4%; I^2^ = 0%; Figure S3B), the cumulative endoleak type Ic/IIIc was 0.2% (95% CI 0–77%; I^2^ = 0%; Figure S3C) and the estimated celiac trunk reintervention rate was 1% (95% CI 0–8%; I^2^ = 0%; Figure S3D).

Regarding the SMA, the estimated instability rate was 1% (95% CI 0–5%; I^2^ = 14%; Figure S4A), the cumulative occlusion/stenosis rate was 0.1% (95% CI 0–34%; I^2^ = 0%; Figure S4B), the cumulative endoleak Ic/IIIc rate was 1% (95% CI 0–3%; I^2^ = 0%; Figure S4C) and the estimated reintervention rate was 1% (95% CI 0–10%; I^2^ = 0%; Figure S4D).

Renal artery instability rate was 4% (95% CI 2–7%; I^2^ = 40%; Figure S5A). A cumulative occlusion/stenosis rate of 2% (95% CI 1–7%; I^2^ = 17%; Figure S5B) and a cumulative endoleak Ic/IIIc rate of 1% (95% CI 0–4%; I^2^ = 0%; Figure S5C) was estimated. The renal artery reintervention rate was 3% (95% CI 2–7%; I^2^ = 0%; Figure S5D).

#### 3.3.5. Follow-Up Outcomes for Vessels Targeted with a Fenestration

A sensitivity analysis for vessels targeted with a fenestration was performed. Data were available and extractable in three studies for technical success [11,20,21] and in five studies for the rest of the outcomes [8,11,20,21,24]. The estimated technical success was 99% (95% CI 92–100%; I^2^ = 7%; Figure S6A). The estimated target vessel instability was 3% (95% CI 1–7%; I^2^ = 60%; Figure S6B). The cumulative occlusion/stenosis rate was 1% (95% CI 0–7%; I^2^ = 36%; Figure S6C) and the endoleak Ic/IIIc rate was 1% (95% CI 0–4%; I^2^ = 31%; Figure S6D). The reintervention rate was estimated at 2% (95% CI 1–5%; I^2^ = 32%; Figure S6E).

## 4. Discussion

This systematic review and proportional meta-analysis demonstrated favorable results regarding both early and midterm target vessel-related outcomes of the BeGraft peripheral and BeGraft peripheral PLUS balloon-expandable covered stents when used as BCS for reno-visceral target vessels in patients managed with f/bEVAR. As up to recently no BCS was commercially approved for f/bEVAR, these results highlight their high competence among the available alternatives [25,26,27]. Similar findings were found in the fEVAR sensitivity analysis, with a highly acceptable rate of patency and low adverse event rates during follow-up, while the recent BeGraft peripheral CE approval for use in fEVAR, along with the newly introduced balloon-expandable covered stent BeFlared (Bentley InnoMed GmbH, Hechingen, Germany), a dedicated BCS for fEVAR procedures, has been a substantiation of BeGraft performance in complex aortic endovascular procedures [28].

The latest available guidelines recommend the application of f/bEVAR as first-line treatment in patients with complex aortic aneurysms and high surgical risk [29,30]. However, both the European Society of Vascular Surgery and American Heart Association guidelines do not specifically and in depth address the issue of BCS choice [29,30]. Preference towards balloon-expandable covered stents in fenestrated and self-expanding covered stents in branched endovascular repair is stated; although strong evidence supports this segregation [4,31], comprehensive cumulative evidence questions the clarity of self-expanding covered stent superiority in bEVAR [32]. Apart from technical- and material-related aspects, antithrombotic treatment choice is also addressed, demonstrating a preference shift from single towards double antiplatelet treatment, though involving case selection based on patency failure risk [29,30], emerging evidence further fortifies this shift [33].

Target vessel-related technical success was achieved in 99% of the target vessels for both f/bEVAR and the fEVAR subgroup. These results stand at the highest levels among previous cohorts, including either multiple different stents or specific alternative balloon-expandable stents [25,26,27,32,34,35]. However, technical success reporting inconsistency was noted among the included studies. Technical success was previously defined only at a patient level and a target vessel-related definition was introduced in the latest reporting standards [16,36,37]. However, even studies published later on demonstrate a remarkable lack of conformity on reporting standard’s definitions regarding target vessel-related technical success, either by not directly implementing or by inadequately interpreting the available definition. Technical success definitions, technical failure events and adjunctive procedures that are presented in Table 3 assist the demonstration of between-study reporting discrepancies.

Regarding the 30-day outcomes, the results of the total cohort were highly acceptable, with target vessel instability, occlusion/stenosis and endoleak rates approaching 0% and a reintervention rate of 1%. Two reinterventions were considered preventive due to a short sealing zone and were performed to avoid future endoleaks, so they were not considered cases of target vessel instability [7,16]. The results are comparable to those of other cohorts reporting on alternative balloon-expandable stents, indicating that major available options have a similar performance profile during the 30-day period demonstrating low adverse event rates [38,39].

Target vessel instability was estimated at 3% during follow-up, accompanied by a reintervention rate of 2% and highly competent endoleak and occlusion rates of 1% for the total cohort of f/bEVAR; the same rates were estimated through the fEVAR sensitivity analysis. These results can be characterized comparable to previous evidence on balloon-expandable covered stent choices’ performance in f/bEVAR procedures [25,26,27]. The positive results may be attributed to the fact that the majority of target vessels in the present study were bridged through fenestrations; prior evidence supports worse target vessel instability and patency rate, as well as reintervention rate in branches compared to fenestrations [35,40]. Endoleak rate was preserved at low levels, demarcating the improvement of the technical features of the current second-generation BeGraft peripheral stents from the first generation, which was related to fractures and subsequent type III endoleaks [5,41].

The vast majority of BeGraft peripheral PLUS stents have been used in branched endovascular aortic repair, owing to their specific characteristics, providing a significant stability when used to bridge target vessels in extensive thoracoabdominal aneurysms [22]. The double sandwich design providing high radial strength combined with the recoil resistance of the cobalt chromium alloy provide appropriate characteristics to achieve adequate distal landing zone apposition and confront high angulation, a factor that is considered responsible for endoleak and occlusion-kinking [5,7,22,42]. Nevertheless, Katsargyris et al. [22] report that almost half of the endoleaks were attributed to type 1c endoleaks in post-dissection thoracoabdominal aneurysms, confirming previous findings of higher target vessel instability in chronic dissections managed with bEVAR [43,44].

Regarding the meta-analyses per type of target vessel, celiac trunk and superior mesenteric artery demonstrated very low rates of instability, endoleak, occlusion/stenosis and reintervention, ranging between 0 and 1%. Renal arteries were associated with higher rates of target vessel adverse events, with a target vessel instability rate of 4% and a reintervention rate of 3%. This finding conforms with the available literature, indicating greater patency loss of renal arteries, compared to visceral ones when targeted during f/bEVAR; an outcome mainly attributed to their smaller diameter and more hostile anatomy [32,34]. Notably, renal artery reintervention rate was relatively high compared to previous cohorts [32] demonstrating a tendency in included cohorts to restore renal artery patency, even if not considered vital as in visceral vessels.

### Limitations

This systematic review and meta-analysis included eight observational cohort studies, seven of which had a retrospective design. Risk of bias was considered serious in seven out of eight studies setting an accountable limitation in results interpretation. The prespecified eligibility criteria concerning the minimum number of treated patients per study, as well as the minimum number of BeGraft stents reported per study, may have affected the findings. Variability in technical success definitions and interpretation of definitions led to inhomogeneous reporting between included studies and this may have affected cumulative results. Follow-up periods among included studies varied importantly. As BCS selection relies on surgeon-driven criteria, this could have introduced important bias. Publication bias assessment and presentation of funnel plots was not performed due to the number of included studies. The low number of events and included studies, along with the nature of the analysis, should indicate conservative interpretation of between-study heterogeneity, as it is quantified by the I^2^ values [17]. Data unavailability separately for the BeGraft peripheral and BeGraft peripheral PLUS, [22] did not allow for a subgroup analysis and/or comparison, constituting an important limitation of this study. Comparative analyses regarding the performance of the BeGraft BCS in fenestrations versus branches were not feasible due to the limited number of studies reporting on bEVAR and unextractable data of mixed f/bEVAR cohorts [22]. However, a sensitivity analysis regarding BeGraft’s behavior in fenestrations was considered feasible and performed, showing low adverse event rates. Scarce availability of data regarding urgent/emergent cases did not allow for a separate analysis. Lack of extractability of data regarding BeGraft stents from other studies that included such kind of stents led to possible bias introduction.

## 5. Conclusions

BeGraft peripheral and BeGraft peripheral PLUS balloon-expandable covered stents have demonstrated excellent outcomes with low target vessel instability, occlusion/stenosis, endoleak and reintervention rates for both 30-day and follow-up periods. For fenestrations, the target vessel instability and reintervention rates were highly acceptable. Target vessel-specific analyses demonstrated similarly low rates of adverse events for the visceral arteries when bridged with the BeGraft stents, with renal artery adverse event rates being slightly higher. The results should be cautiously interpreted due to the serious risk of bias in most of the included studies and the very low quality of evidence.

## Figures and Tables

**Table 1:** Main characteristics of included studies.

Author	Year	Journal	Study Period	Type of Intervention	Device Platform	Available Follow-Up (Months)
Becker, et al. [11]	2024	J Endovasc Ther	September 2018–December 2022	CM fEVAR/PM fEVAR	Zenith platform (CM), Cook (PM)	20 (range 6–32)
Clough, et al. [20]	2022	J Endovasc Ther	November 2015–September 2016	CM fEVAR	Zenith platform	33 (IQR 17–36)
D’Oria, et al. [21]	2023	J Endovasc Ther	2015–2021	CM fEVAR	Zenith Platform, Anaconda Platform	19.8 ±13.2
Abisi, et al. [7]	2021	J Endovasc Ther	May 2017–May 2020	CM/OTS bEVAR	Zenith platform (CM), T-branch/E-nside (OTS)	15 (range 4–36)
Katsargyris, et al. [22]	2024	Eur J Vasc Endovasc Surg	January 2018– July 2023	CM fEVAR/CM fbEVAR	Zenith platform	17 (range 0–72)
Maurel, et al. [23]	2017	J Vasc Surg	October 2015– March 2016	CM fEVAR	Zenith platform	N.A.
Tran, et al. [8]	2021	J Vasc Surg	September 2019– April 2020	CM fEVAR	Zenith platform	6.3 ± 3.2
Mancuso, et al. [24]	2023	J Endovasc Ther	January 2013– December 2021	CM fEVAR	Anaconda platform	35 (IQR 10–63)

CM: custom-made; PM: physician-modified; OTS: off-the-shelf; fEVAR: fenestrated endovascular aortic repair; bEVAR: branched endovascular aortic repair; fbEVAR: fenestrated and branched endovascular aortic repair; N.A.: not applicable; follow-up time is expressed in mean ± standard deviation or median (range) or median (IQR; interquartile range).

**Table 2:** The number of patients, target vessels and stents and the type of aneurysm of included patients.

Author	Patients	TVs	BCS	BeGraft Peripheral/ BeGraft Peripheral PLUS Stents			Type of Aneurysm		
				ALL	B	B+	Juxta-Para- Renal	TAAA	Failed EVAR
Becker, et al. [11]	113	361	361	361	361	0	Juxta: 68, Para: 14	I: 6, II: 10, III: 5, IV: 8, V: 5	N.A.
Clough, et al. [20]	39	101	101	101	101	0	Juxta: 9, Para: 10	I: 2, II: 3, III: 2, IV: 3, V: 4	6
D’Oria, et al. [21]	82	266	266	266	266	0	72	10	N.A.
Abisi, et al. [7]	46	163	163	163	0	163	Para: 6	I: 1, II: 10, III: 17, IV: 12, V: 0	N.A.
Katsargyris, et al. [22]	281	765	816	816	532	284	Para: 116	I: 21, II: 21, III: 35, IV: 68, V: 5	N.A.
Maurel, et al. [23]	30	58	56	10	10	0	TAAA I-III: 8, TAAA IV and Para: 19		
Tran, et al. [8]	12	46	46	46	46	0	0	I: 0, II: 4, III: 3, IV: 5, V: 0	N.A.
Mancuso, et al. [24]	75	226	241	28	13	15	Juxta: 75	0	N.A.

TV: target vessel; TAAA: thoracoabdominal aortic aneurysm; EVAR: endovascular aortic repair; N.A.: not available.

**Table 3:** Technical success definition and reported intraoperative adjunctive procedures per included study.

Study	Technical Success Definition	Reported Technical Failures	TV Related Adjunctive Intraoperative Procedures
Becker, et al. [11]	Bridging stent technical success: placement of the bridging stent in the planned position	Two TVs: (1) impossible cannulation leading to coiling, (2) impossible stent advancement (ostial stenosis)	Twelve target vessels relined for kinking, tortuosity, residual dissection
Spear, et al. [9]	N.A.	No technical failure	Two additional angioplasties, two additional covered stent, one additional nitinol stent for acute angulation in distal landing zone, one stent kinking (implantation of an additional BeGraft)
D’ Oria, et al. [21]	(According to current reporting standards^15^)	5 TVs: 1 CT angioplasty for ostial stenosis, 2 RA re-flaring, 1 RA relining and 1 angioplasty for ostial stenosis	Reported technical failures + RA branch embolization due to kidney hematoma
Abisi, et al. [7]	Safe delivery and precise deployment of the BeGraft peripheral plus stent graft, in addition to target vessel patency	1 TV: faulty balloon led to partial expansion of stent that needed re-flaring	Thirty TVs needed distal or proximal extension with BECS, two TVs needed distal extension with SEBMS for dissection and three TVs needed distal extension with SEBMS for excessive angulation
Katsargyris, et al. [22]	Successful catheterization and stent placement in all intended TVs (based on current reporting standards^15^)	4 TVs: (1) BeGraft peripheral plus impossible stent implantation in the SMA, (2) renal artery rupture during balloon inflation and placement of two additional covered stents, (3) renal artery dissection, two SEBMS implantation (4) stent disconnection due to sheath advancement into the target vessel- BECS proximally	Reported technical failures + 18 relinings to smooth distal landing, one relining for dissection, one additional BECS deployment due to insufficient overlap
Maurel, et al. [23]	Successful introduction and deployment of the device in the absence of surgical conversion or mortality, successful stenting of all fenestrations intended in the surgeon’s plan, the absence of type I endoleak or obstruction of branch vessels on completion angiography and survival through 24 h (per case) (based on previous reporting standards^36^)	One case with ileal perforation (not TV related)One case with SMA dissection with distal malperfusion, reintervention and stenting	Additional CT and SMA ballooning for flaring, additional renal stenting for dissection, additional renal stenting for type IIIc endoleak
Tran, et al. [8]	NA	No technical failures	NA
Mancuso, et al. [24]	NA	Three TVs where no catheterization and consequent stent implantation was achieved	Embolization due to persistent Ic endoleak and inability to re-catheterize; stent kinking; relining; stent dislodgement–replacement; fracture after flaring; additional stent deployment; stent dislodgement into the sheath after flaring; replacement

TV: target vessel; N.A.: not available; CT: celiac trunk; RA: renal artery; BECS: balloon-expandable covered stent; SEBMS: self-expanding bare metal stent; SMA: superior mesenteric artery.

## Data Availability

The data supporting the conclusions of this article will be made available upon request from the corresponding author.

## Supplementary Materials

The following supporting information can be downloaded at https://www.mdpi.com/article/10.3390/jcm14155221/s1, Figure S1: Risk of bias assessment with ROBINS-1 tool of individual studies included in the systematic review and meta-analysis [7,8,11,20,21,22,23,24]; Figure S2: Forest plot for results on 30-day outcomes: (A) Target-vessel instability, (B) Occlusion/ stenosis rate, (C) Endoleak Ic/ IIIc rate, (D) Reintervention rate [7,11,20,21,23]; Figure S3: Forest plot for celiac trunk specific results on follow-up outcomes: (A) Target-vessel instability, (B) Occlusion/ stenosis rate, (C) Endoleak Ic/ IIIc rate, (D) Reintervention rate [7,8,11,20,21,22]; Figure S4: Forest plot for superior mesenteric artery specific results on follow-up outcomes: (A) Target-vessel instability, (B) Occlusion/ stenosis rate, (C) Endoleak Ic/ IIIc rate, (D) Reintervention rate [7,8,11,20,21,22]; Figure S5: Forest plot for renal artery specific results on follow-up outcomes: (A) Target-vessel instability, (B) Occlusion/ stenosis rate, (C) Endoleak Ic/ IIIc rate, D) Reintervention rate [7,8,11,20,21,22]; Figure S6: Forest plot of primary outcomes in vessels targeted through a fenestration: (A) Technical success (B) Target-vessel instability, (C) Occlusion/ stenosis rate, (D) Endoleak Ic/ IIIc rate, (E) Reintervention rate [8,11,20,21,24]; Table S1: The PICO format of the systematic review and meta-analysis; Table S2: Search strategy of the systematic review and meta-analysis; Table S3: Quality assessment, based on GRADE approach [7,8,11,20,21,22,24]; Table S4: Summary of evidence based on GRADE assessment [7,8,11,20,21,22,24].

## Abbreviations

The following abbreviations are used in this manuscript:

f/bEVAR	Fenestrated/branched endovascular aortic repair
BCS	Bridging covered stent
CI	Confidence interval
CM	Custom-made
PM	Physician-modified
OTS	Off-the-shelf
TAAA	Thoracoabdominal aortic aneurysm
EVAR	Endovascular aortic repair
TV	Target vessel
CT	Celiac trunk
SMA	Superior mesenteric artery
RA	Renal artery
BECS	Balloon-expandable covered stent
SEBMS	Self-expanding bare metal stent
N.A.	Not applicable/ Not available
IQR	Interquartile range
