# Supplementary material for: Systematic Review and Meta-Analysis on the BeGraft Peripheral and BeGraft Peripheral PLUS Outcomes as Bridging Covered Stents in Fenestrated and Branched Endovascular Aortic Repair
Source: J Clin Med. 2025 Jul 23;14(15):5221. doi: 10.3390/jcm14155221 (PMC12347551; doi:10.3390/jcm14155221)
Supplement: Supplementary file 1 [file jcm-14-05221-s001.zip › Supplementary material.v10.pdf]

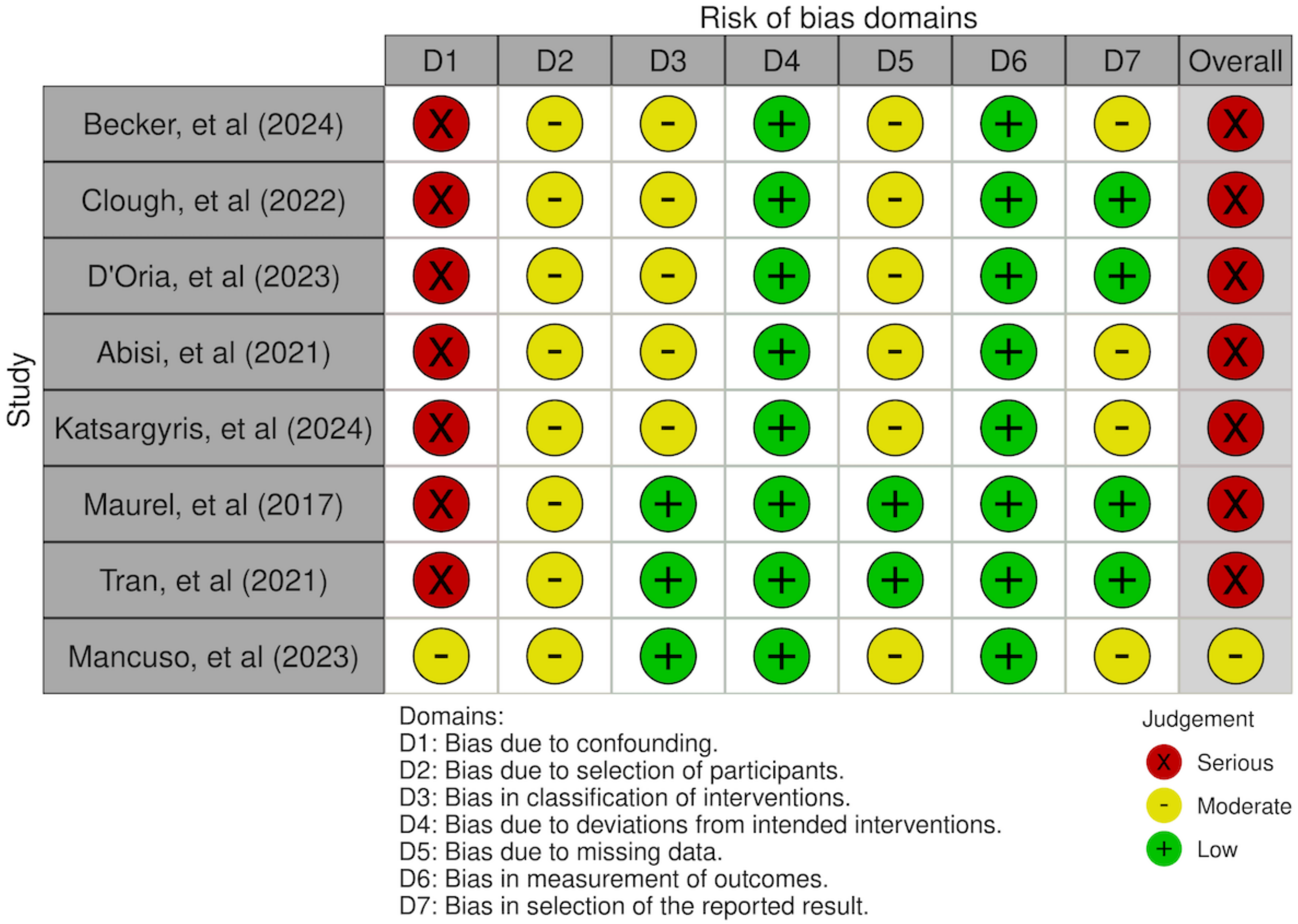

Supplementary figure S1. Risk of bias assessment with ROBINS-1 tool of individual studies included in the systematic review and meta-analysis [7,8,11,20-24].

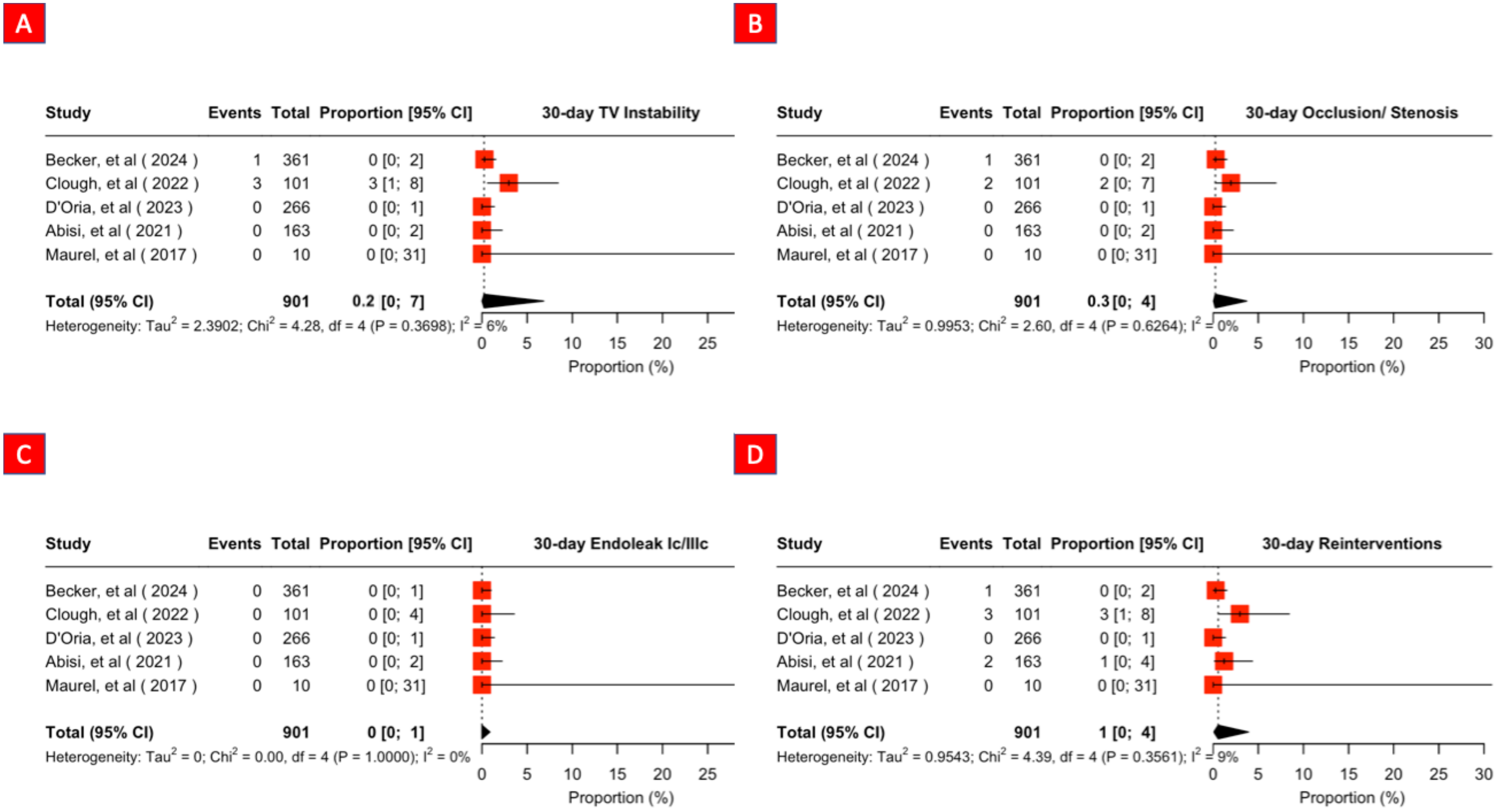

Supplementary figure S2. Forest plot for results on 30-day outcomes: A) Target-vessel instability, B) Occlusion/ stenosis rate, C) Endoleak Ic/ IIIc rate, D) Reintervention rate [7,11,20,21,23].

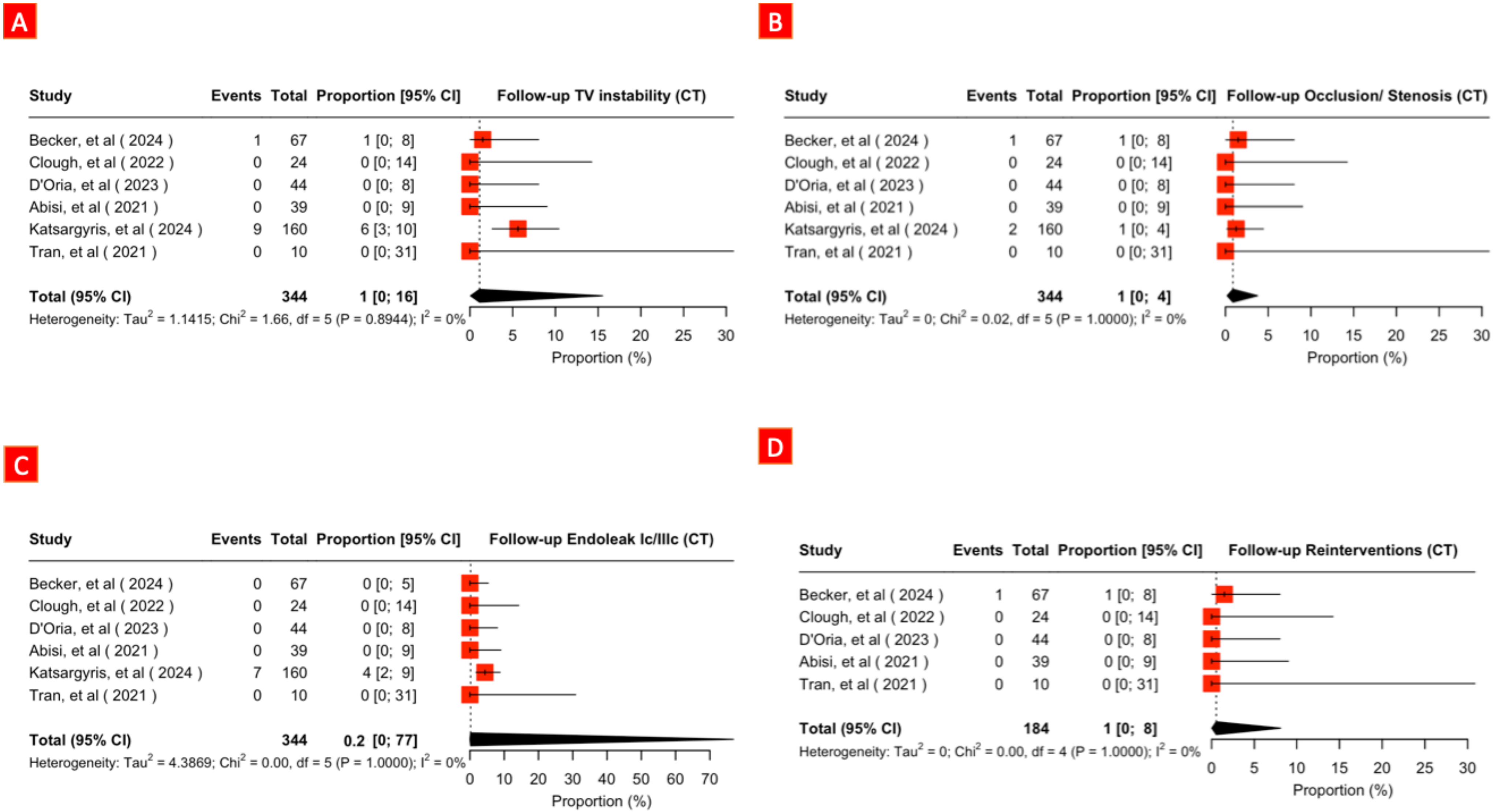

Supplementary figure S3. Forest plot for celiac trunk specific results on follow-up outcomes: A) Target-vessel instability, B) Occlusion/ stenosis rate, C) Endoleak Ic/ IIIc rate, D) Reintervention rate [7,8,11,20-22].

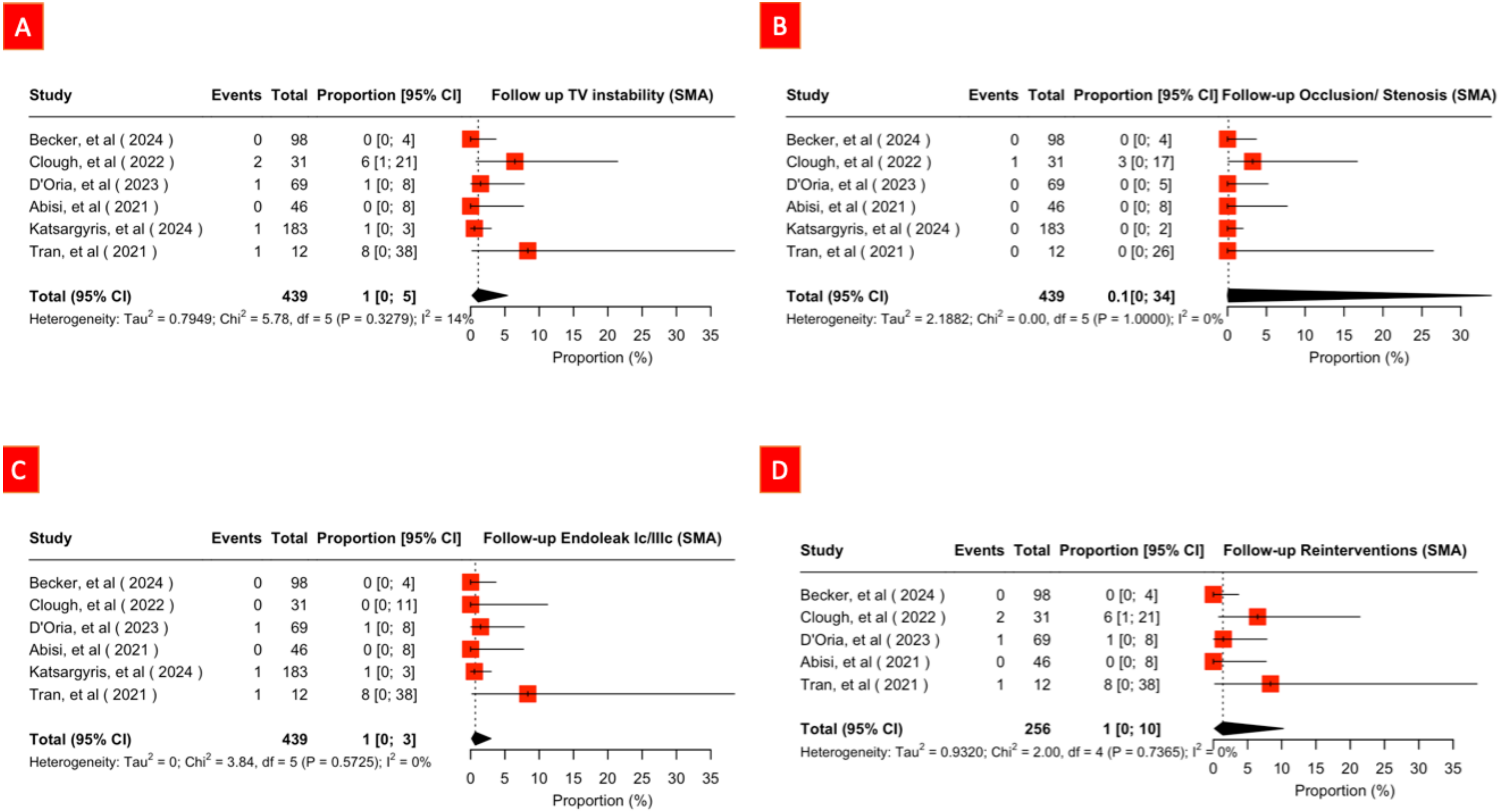

Supplementary figure S4. Forest plot for superior mesenteric artery specific results on follow-up outcomes: A) Target-vessel instability, B) Occlusion/ stenosis rate, C) Endoleak Ic/ IIIc rate, D) Reintervention rate [7,8,11,20-22].

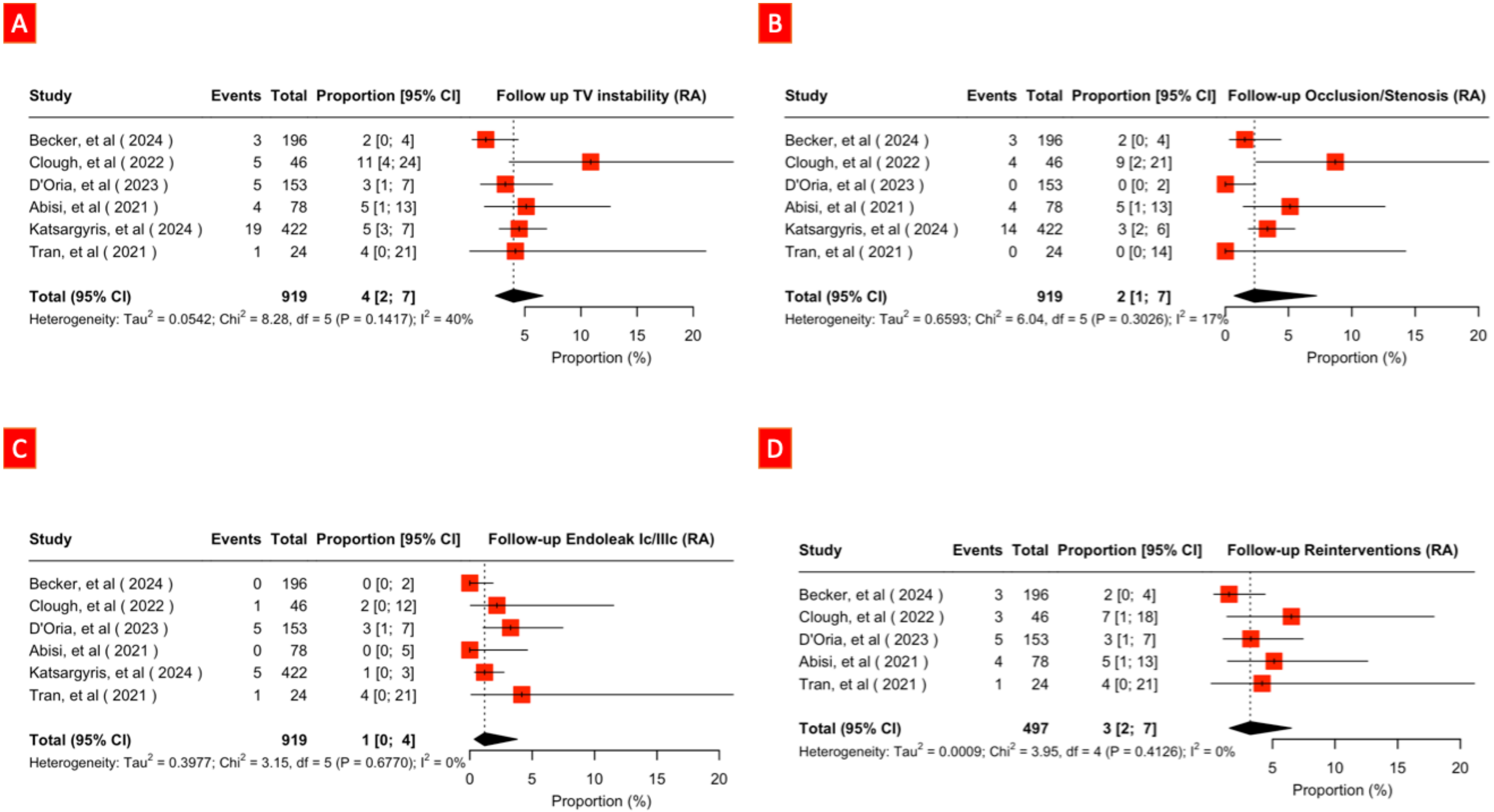

Supplementary figure S5. Forest plot for renal artery specific results on follow-up outcomes: A) Target-vessel instability, B) Occlusion/ stenosis rate, C) Endoleak Ic/ IIIc rate, D) Reintervention rate [7,8,11,20-22].

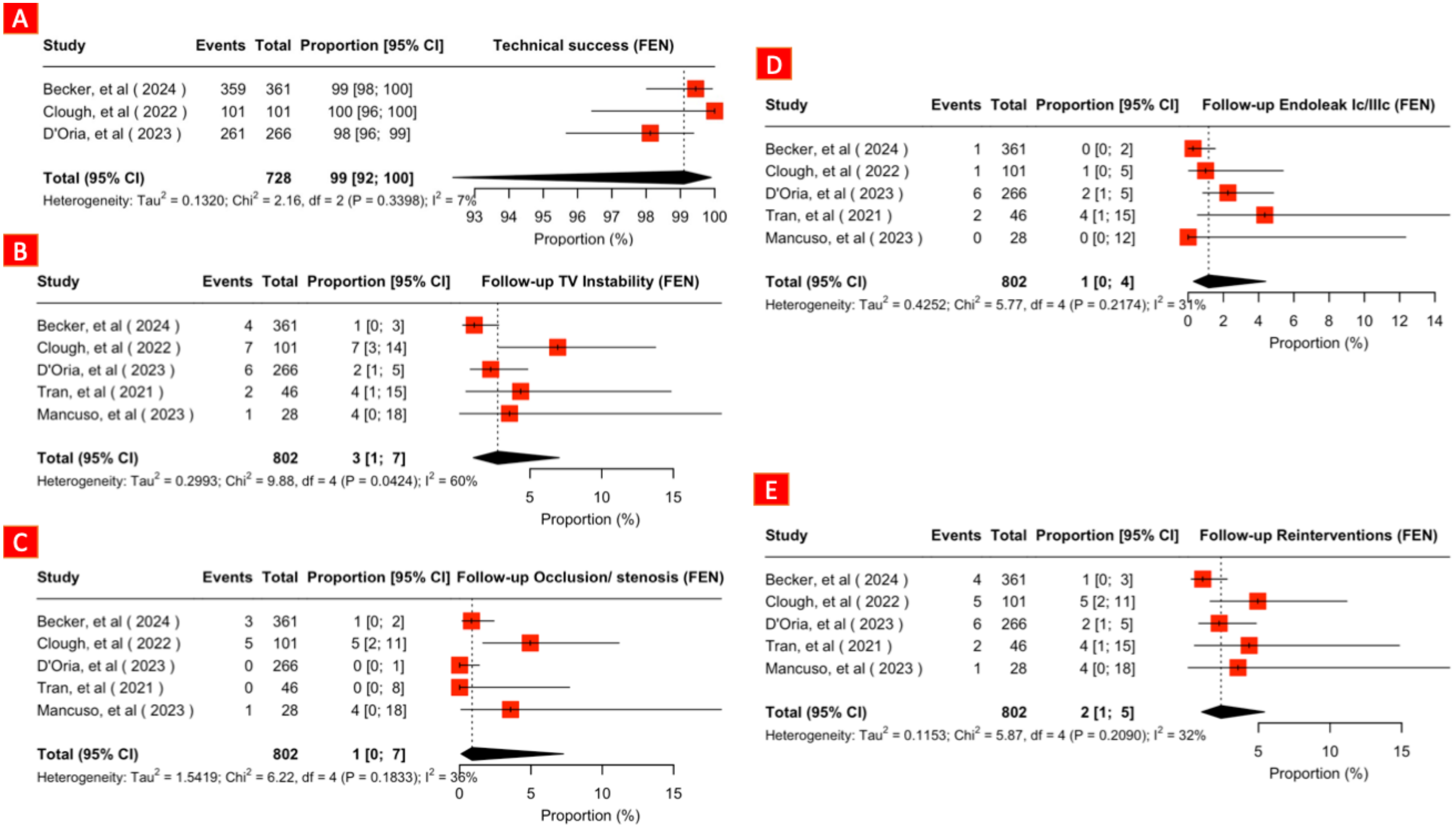

Supplementary figure S6. Forest plot of primary outcomes in vessels targeted through a fenestration: A) Technical success B) Target-vessel instability, C) Occlusion/ stenosis rate, D) Endoleak Ic/ IIIc rate, E) Reintervention rate [8,11,20,21,24].

P	Patient, population or problem	Patients with complex aortic aneurysms (juxtarenal, pararenal, thoracoabdominal)
I	Intervention, prognostic factor or exposure	Fenestrated or branched endovascular aortic repair where the BeGraft peripheral/ BeGraft peripheral PLUS balloon-expandable covered stent was used as bridging stent
C	Comparison of intervention	-
O	Outcome to be measured	Technical success, target vessel instability rate, occlusion/stenosis rate, endoleak (type Ic/ IIIc) rate, reintervention rate
	What type of question is asked?	What is the proportion of target vessel related adverse events when the BeGraft peripheral/ BeGraft peripheral PLUS balloon-expandable covered stents were used as bridging stents during fenestrated/ branched endovascular aortic repair?
T	Type of studies to be included	Observational, trials (randomized, non-randomized)

Supplementary table S1. The PICO format of the systematic review and meta-analysis.

Frame	Search terms (used both as full text and "MeSH" terms)	Search	Inclusion criteria	Exclusion criteria	Sources
P (patients, participants, population)	#1 "thoracoabdominal aortic aneurysm",<br>#2 "juxtarenal aortic aneurysm",<br>#3 "pararenal aortic aneurysm"	#1 OR #2 OR #3 OR AND #5 OR #6	Observational (retrospective or prospective) and trials (randomized or non-randomized) reporting on BeGraft peripheral/ BeGraft peripheral PLUS performance as a bridging stent in patients managed with fenestrated/branched endovascular aortic repair for complex aortic aneurysms.<br>Studies reporting results on a population where at least ten BeGraft stents were used<br>Peer reviewed journals<br>English language	Irrelevant title<br>Irrelevant full text<br>Non- English<br>Not applicable study type (editorial, reviews, meta-analyses, technical notes, images, case series <10 patients, case reports)<br>Studies reporting on parallel graft technique or surgeon-modified devices.<br>Studies reporting on other types of bridging stents or not stating specific stent used<br>Studies with non-extractable or irrelative data to outcomes of interest regarding BeGraft peripheral/ BeGraft peripheral PLUS	PubMed, Cochrane library, Embase (via Ovid)
I (intervention)	#4 "fenestrated endovascular aortic repair",<br>#5 "branched endovascular aortic repair"				
C (comparator, reference test)	NA				
O (outcome)	Not applied in order not to further limit the sensitivity of our search				
Time	Search period: 2016-2025<br>Last search: 15.04.2025				

Supplementary table S2. Search strategy of the systematic review and meta-analysis. Last search was performed on 15.04.2025. Studies reporting on Begraft peripheral/ Begraft peripheral PLUS used as bridging stents during fenestrated/branched endovascular aortic repair were considered eligible as far as they reported on at least ten of these specific stents and when more than ten patients were included.

Quality assessment							Summary of findings			
							N of target vessels	Effect		Importance
No of studies	Study design	Risk of bias	Inconsistency	Indirectness	Imprecision	Other considerations	N of target vessels	Relative (95% CI)	Quality	
Target- vessel related technical success in patients managed with f/bEVAR; assessed as technical success related to target vessels bridged with BeGraft peripheral and/or BeGraft peripheral PLUS balloon-expandable covered stent										
5 [7,11,20-22]	5 retrospective [7,11,20-22] observational studies	Serious	Not serious	Not serious	Not serious	None	1656	99% (98-100%)	Very low	Critical
Target- vessel instability in patients managed with f/bEVAR; assessed as instability related to target vessels bridged with BeGraft peripheral and/or BeGraft peripheral PLUS balloon-expandable covered stent										
7 [7,8,11,20-22,24]	6 retrospective [7,11,20-22,24], 1 prospective [8] observational studies	Serious	Moderate	Not serious	Not serious	None	1730	3% (2-5%)	Very low	Critical
Target- vessel related occlusion/ stenosis in patients managed with f/bEVAR; assessed as occlusions/ stenoses related to target vessels bridged with BeGraft peripheral and/or BeGraft peripheral PLUS balloon-expandable covered stent										
7 [7,8,11,20-22,24]	6 retrospective [7,11,20-22,24], 1 prospective [8] observational studies	Serious	Not serious	Not serious	Not serious	None	1730	1% (1-4%)	Very low	Important
Endoleak type Ic/ IIIc in patients managed with f/bEVAR; assessed as endoleak type Ic or IIIc related to target vessels bridged with BeGraft peripheral and/or BeGraft peripheral PLUS balloon-expandable covered stent										
7 [7,8,11,20-22,24]	6 retrospective [7,11,20-22,24], 1 prospective [8] observational studies	Serious	Not serious	Not serious	Not serious	None	1730	1% (0-3%)	Very low	Important
Target- vessel related reintervention in patients managed with f/bEVAR; assessed as reinterventions related to target vessels bridged with BeGraft peripheral and/or BeGraft peripheral PLUS balloon-expandable covered stent										
7 [7,8,11,20-22,24]	6 retrospective [7,11,20-22,24], 1 prospective [8] observational studies	Serious	Not serious	Not serious	Not serious	None	1730	2% (2-4%)	Very low	Important

Supplementary table S3. Quality assessment, based on GRADE approach [7,8,11,20-22,24].

Outcomes	Number of target vessels, number of studies, follow-up	Quality of evidence (GRADE)	Relative effect (95% CI)	Conclusions	Recommendations
Target vessel related technical success in patients managed with f/bEVAR	1656 TVs, 5 retrospective [7,11,20-22] observational studies	Very low	99% (98-100%)	There is a small body of very low quality of evidence suggesting that BeGraft peripheral and/or Begraft peripheral PLUS is related to 99% rate of technical success	Further evidence from high quality studies is needed
Target vessel instability in patients managed with f/bEVAR during follow-up	1730 TVs, 6 retrospective [7,11,20-22,24], 1 prospective [8] observational studies, mid-term follow-up	Very low	3% (2-5%)	There is a small body of very low quality of evidence suggesting that BeGraft peripheral and/or Begraft peripheral PLUS is related to 3% rate of target vessel instability during midterm follow-up	Further evidence from high quality studies is needed
Target vessel related occlusions/ stenoses in patients managed with f/bEVAR during follow-up	1730 TVs, 6 retrospective [7,11,20-22,24], 1 prospective [8] observational studies, mid-term follow-up	Very low	1% (1-4%)	There is a small body of very low quality of evidence suggesting that BeGraft peripheral and/or Begraft peripheral PLUS is related to 1% rate of occlusion/ stenosis during mid-term follow-up	Further evidence from high quality studies is needed
Target vessel related endoleak type Ic or IIIc in patients managed with f/bEVAR during follow-up	1730 TVs, 6 retrospective [7,11,20-22,24], 1 prospective [8] observational studies, mid-term follow-up	Very low	1% (0-3%)	There is a small body of very low quality of evidence suggesting that BeGraft peripheral and/or Begraft peripheral PLUS is related to 1% rate of endoleak type Ic/ IIIc during mid-term follow-up	Further evidence from high quality studies is needed
Target vessel related reinterventions in patients managed with f/bEVAR during follow-up	1730 TVs, 6 retrospective [7,11,20-22,24], 1 prospective [8] observational studies, mid-term follow-up	Very low	2% (2-4%)	There is a small body of very low quality of evidence suggesting that BeGraft peripheral and/or Begraft peripheral PLUS is related to 2% rate of reintervention during mid-term follow-up	Further evidence from high quality studies is needed

Supplementary table S4. Summary of evidence based on GRADE assessment [7,8,11,20-22,24].
